# Boosting the Supercapacitance of Nitrogen‐Doped Carbon by Tuning Surface Functionalities

**DOI:** 10.1002/cssc.201700902

**Published:** 2017-08-15

**Authors:** Jasper Biemolt, Ilse M. Denekamp, Thierry K. Slot, Gadi Rothenberg, David Eisenberg

**Affiliations:** ^1^ Van't Hoff Institute for Molecular Sciences University of Amsterdam Science Park 904 Amsterdam 1098 XH The Netherlands; ^2^ Current address: Schulich Faculty of Chemistry Technion—Israel Institute of Technology Haifa 3200003 Israel

**Keywords:** carbon, electrochemical capacitors, electrochemistry, nitrogen doping, surface chemistry

## Abstract

The specific capacitance of a highly porous, nitrogen‐doped carbon is nearly tripled by orthogonal optimization of the microstructure and surface chemistry. First, the carbons’ hierarchical pore structure and specific surface area were tweaked by controlling the temperature and sequence of the thermal treatments. The best process (pyrolysis at 900 °C, washing, and subsequent annealing at 1000 °C) yielded a carbon with a specific capacitance of 117 F g^−1^—nearly double that of a carbon made by a typical single‐step synthesis at 700 °C. Following the structural optimization, the surface chemistry of the carbons was enriched by applying an oxidation routine based on a mixture of nitric and sulfuric acid in a 1:4 ratio at two different treatment temperatures (0 and 20 °C) and different treatment times. The optimal treatment times were 4 h at 0 °C and only 1 h at 20 °C. Overall, the specific capacitance nearly tripled relative to the original carbon, reaching 168 F g^−1^. The inherent nitrogen doping of the carbon comes into interplay with the acid‐induced surface functionalization, creating a mixture of oxygen‐ and nitrogen‐oxygen functionalities. The evolution of the surface chemistry was carefully followed by X‐ray photoelectron spectroscopy and by N_2_ sorption porosimetry, revealing stepwise surface functionalization and simultaneous carbon etching. Overall, these processes are responsible for the peak‐shaped capacitance trends in the carbons.

## Introduction

Moving to sustainable energy sources requires efficient energy storage solutions.^1^ Electrochemical devices such as batteries, fuel cells, and supercapacitors play a central role in this field.^2^, ^3^, ^4^ Each renewable energy source (e.g., wind, solar, geothermal) sets its own requirements for energy density, power density, life‐time, cost, and size. Supercapacitors (also called electrochemical capacitors or ultracapacitors) are important power sources for applications that require fast charge and discharge.^5^, ^6^, ^7^, ^8^, ^9^ For example, the transport industry uses supercapacitors for regenerative braking and for boosting power delivery alongside batteries.^10^ In addition to their high power density, supercapacitors offer excellent reversibility (typically ≈10^6^ cycles) and safer use and production than batteries. However, the energy density of supercapacitors is 2–3 orders of magnitude lower than that of batteries.

Supercapacitors operate through two fundamental mechanisms. The first is electric double layer capacitance (EDLC), in which charging the electrode leads to adsorption and desorption of electrolyte counter‐ions at its surface. This sorption is fast and reversible, determining the high‐power density of the device and its longevity. The second mechanism is pseudocapacitance (PC), wherein fast Faradaic reactions occur at the surface, storing charge in chemical bonds and boosting the energy density.^11^, ^12^ These redox reactions are fast enough so that diffusion limitations are small and power density remains high. PC was reported for oxides such as MnO_2_ 13 and Nb_2_O_5_,^14^ as well as in conducting polymers (e.g., polyaniline).^15^ However, these materials suffer from limitations such as low conductivity and stability as well as high cost.

**Figure 1:**
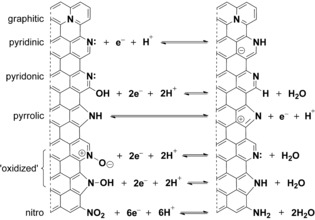
Types of nitrogen functionalities occurring in nitrogen‐doped carbon and the pseudocapacitive reactions they might participate in.^6^, ^7^, ^8^, ^9^, ^19^, ^20^, ^21^, ^22^, ^23^

Porous carbon, with its high surface area, high conductivity, and low cost, is the most popular material for EDLC supercapacitors.^16^, ^17^, ^18^ Furthermore, doping carbon with O or N atoms creates active sites for pseudocapacitive reactions (Figure 1). Dopants can be introduced either during synthesis (e.g., by pyrolysis of biomass^24^ or of nitrogen‐containing salts)^7^, ^25^, ^26^, ^27^, ^28^ or by postpyrolysis surface treatment. Surface functionalization decouples the carbon bulk structure from surface chemistry. This allows for orthogonal optimization and for a better understanding of pseudocapacitive active sites. Surface treatments vary in complexity from brute‐force boiling in acid^29^ to grafting of predesigned functional groups.^30^ The simpler treatments are more practical; however, their effect on supercapacitance is more complex.^31^, ^32^ Functional groups can affect conductivity, wettability, exposed surface area, and stability.

We recently reported a new family of carbons^25^, ^26^, ^27^, ^28^ for which hierarchical porosity, high nitrogen doping, and conductivity suggested they may also be useful as supercapacitors.^33^ These carbons were prepared by pyrolyzing highly crystalline magnesium‐nitrilotriacetate metal–organic frameworks (MOFs). The robust and sustainable synthesis method uses safe and abundant materials and is performed on a multigram scale.

We now report that the capacitance of such carbons is nearly tripled by orthogonally optimizing the carbon structure and the surface chemistry. First, we studied the microstructure and doping of the bulk carbon by tweaking the temperature and sequence of the thermal treatments. Second, we functionalized the surface by a simple yet powerful treatment in mineral acids. The surface functionalities evolve with time and temperature of the oxidative treatments. We found that the pre‐existing nitrogen dopants play an important role in the formation and pseudocapacitive contribution of the new surface functionalities.

## Results and Discussion

### Capacitance and microstructure

Supercapacitor carbons are often produced by pyrolysis at temperatures of approximately 700 °C. This temperature is high enough to carbonize the precursors yet low enough to conserve the heterodopants. Pyrolysis of magnesium nitrilotriacetic acid (MgNTA) at 700 °C yields a carbon with high specific surface area (SSA; 830 m^2^ g^−1^) and high nitrogen content (11 at %), yet moderate specific capacitance (62 F g^−1^ in 1 m H_2_SO_4_ at 5 mV s^−1^). The low capacitance may result from insufficient micropore volume (0.27 mL g^−1^, out of a total pore volume of 1.05 mL g^−1^), from low conductivity, or from a small number of pseudocapacitive sites. The pyrolysis temperature was increased up to 1000 °C (samples denoted NC‐700 to NC‐1000, Table 1). Both the micropore volume and SSA increased to 0.54 mL g^−1^ and 1606 m^2^ g^−1^, respectively, when the samples were pyrolyzed at 900 °C. This increase was attributed to the evaporation of tar from nanometric interstices between graphene sheets.^27^ As a result, the carbon's specific capacitance was boosted by 55 % up to 96 F g^−1^.

**Table 1:** Effect of heat treatments on carbon properties.

Carbon sample^[a]^	SSA^[b]^	*V*_micro_^[c]^	*V*_total_^[d]^	Atomic fraction^[e]^ [at %]			*C*_sp_^[f]^
	[m^2^ g^−1^]	[cm^3^ g^−1^]	[cm^3^ g^−1^]	C	N	O	[F g^−1^]
NC‐700	830	0.27	1.05	80.5	11.5	8.0	62
NC‐800	1144	0.38	1.68	89.7	5.2	5.1	108
NC‐900	1606	0.54	2.24	86.8	7.4	5.8	96
NC‐1000	1410	0.51	2.47	83.7	11.0	5.3	118
NC‐700*	928	0.33	1.20	91.1	6.7	2.2	83
NC‐800*	1286	0.45	1.86	91.6	6.0	2.4	107
NC‐900*	1831	0.61	3.10	91.4	5.9	2.7	117
NC‐1000*	1519	0.55	2.79	94.0	4.0	2.0	96

[a] The number denotes the pyrolysis temperature, and the asterisk marks a second heat treatment at 1000 °C in Ar. [b] Specific surface area as determined by BET2 analysis of N_2_ desorption at 77 K. [c] Total pore volume filled up to a relative pressure (*P*/*P*_0_) of 0.99. [d] Micropore volume calculated by the Dubinin–Radushkevich method. [e] Near‐surface elemental composition obtained from XPS. [f] Specific capacitance measured at a scan rate of 5 mV s^−1^.

The pyrolysis temperature has a complex effect on the SSA, micropore volume, nitrogen content, and specific capacity (Table 1). For example, raising the pyrolysis temperature to 1000 °C causes micropore shrinkage, yet an increase in capacitance. However, heating the carbon to 1000 °C after the MgO had been washed out yielded the best porosity: SSA of 1830 m^2^ g^−1^ and micropore volume of 0.61 mL g^−1^ (3.10 mL g^−1^ total pore volume). Carbons heated in this manner are denoted with an asterisk. Possibly, the removal of MgO eases the clearing of amorphous goo and anneals the surface by rearranging the more labile kinks (a similar effect is seen with mixed oxides).^34^ However, MgO may also stabilize the presence of N content; sample NC‐1000 has 11.0 at % nitrogen, whereas NC‐900* has only 4.1 at % nitrogen.

**Figure 2:**
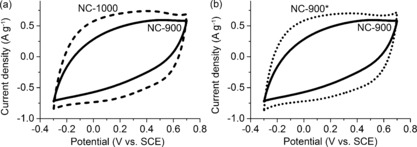
Cyclic voltammetry of nitrogen‐doped carbons after pyrolysis at 900 °C (NC‐900, solid curve) compared to (a) pyrolysis at 1000 °C (NC‐1000, dashed curve), or (b) pyrolysis at 900 °C followed by a heat treatment at 1000 °C (NC‐900*, dotted curve). Measured in 1 m H_2_SO_4_ at 5 mV s^−1^.

The highest capacitance (117–118 F g^−1^) in the heat‐treatment series was obtained from the two carbons heated to 1000 °C (NC‐1000 and NC‐900*). The shape of the voltammograms of these samples is closest to a square shape (Figure 2 and Figure S1 in the Supporting Information). This indicates a fast and homogeneous charging of the carbon electrodes. The fact that these were not necessarily the carbons with the highest SSA emphasizes the importance of other factors. These include conductivity (as determined by degree of graphitization) and surface redox chemistry introducing slower pseudocapacitive reactions.

### Capacitance and surface chemistry

Next, we set out to improve the PC of our carbons. Per definition, PC depends on fast redox reactions between incoming (ionic) species and surface functional groups. Pristine (i.e., undoped) carbon doesn't show any PC, but nitrogen‐doped carbon has PC activity.^29^, ^35^ In our carbons, a hint of redox activity is evident in the broad, reversible “hump” in the cyclic voltammogram (see for example Figure 2 a; NC‐1000).

In our acidic aqueous environment, the most common ionic species are hydronium (H_3_O^+^) ions. Therefore, we tried to create surface sites that would facilitate proton/electron transfer reactions with these ions. To do this, we used a 1:4 mixture of concentrated nitric acid and sulfuric acid. Nitric acid is a strong oxidizing and nitrating agent at low temperatures,^36^, ^37^ especially in the presence of sulfuric acid.^31^ Recently, Lin et al. treated a nitrogen‐doped carbon in hot nitric acid, boosting its PC through an acid–base surface reaction.^29^ This is the only example of such a treatment for a nitrogen‐doped carbon that we know.

We then ran two sets of experiments, at 0 °C and at 20 °C, for up to 18 h. Control experiments showed that longer and hotter runs led to a collapse of the structure. Samples are denoted as NC‐*xx*‐*yy*, where *xx* is the treatment temperature (0 or 20 °C) and *yy* is the treatment time (e.g., 1 h).

**Figure 3:**
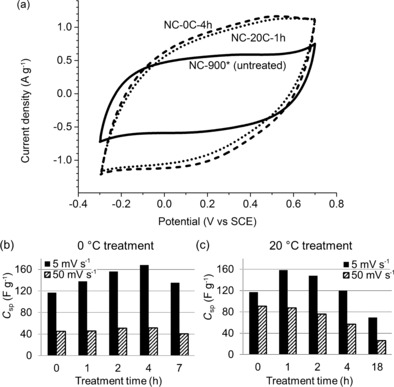
Effect of oxidative surface treatments on supercapacitance. (a) Cyclic voltammetry at 5 mV s^−1^ in 1 m H_2_SO_4_ of the untreated carbon (NC‐900*, solid line), and the best treated carbons NC‐0C‐4h (dashed curve) and NC‐20C‐1h (dotted curve). (b, c) Ultimate increase in specific capacitance as a function of treatment time at treatment temperatures of (b) 0 and (c) 20 °C shown for scan rates of 5 (solid bars) and 50 mV s^−1^ (hatched bars).

Cyclic voltammetry of the carbons in 1 m H_2_SO_4_ showed that the oxidative treatments considerably increased the specific capacitance (Figure 3). First, the voltammogram shapes changed from square to more round. This indicates a slower charge transfer during electrode polarization reversal, which may be related to surface functionalization through several mechanisms. These include the introduction of slow‐charging pseudocapacitive sites, the blocking of micropores, and increased electron localization (lower conductivity). A broad peak appeared at 0.5 V vs. saturated calomel electrode (SCE) after the treatment (Figure 3 a and S3). This reversible peak corresponds to a new redox process occurring at the carbon surface. The width of the peak—which is probably related to a broad distribution of pseudocapacitive surface sites^9^, ^29^—does not allow precise integration.^38^ However, if we rule out contributions by EDLC, we can assign the entire increase to PC and estimate its contribution to total capacitance (see below).

The temperature and time of the treatment have a profound effect on the capacitance (Figure 3 b and c and Table 2). At 0 °C, the capacitance increases with treatment time until it peaks at 4 h. At 20 °C, even 1 h is sufficient to boost the capacitance to its maximum value. The maximum capacitances were 158–168 F g^−1^ at 5 mV s^−1^, an increase of approximately 40 % over the untreated nitrogen‐doped carbon. This carbon retained its activity over several cycles and faster scan rates (Figure S2), including at 10 (133 F g^−1^) and 25 mV s^−1^ (81 F g^−1^). The lower response at faster scan rates corresponds to slower charge–discharge processes similar to the change in the shape of the voltammogram (see above).

**Table 2:** Effect of acidic surface treatments on carbon NC‐900*.

Carbon sample^[a]^	SSA^[b]^	*V*_micro_^[c]^	*V*_total_^[d]^	Atomic fraction^[e]^ [at %]			*C*_sp_^[f]^
	[m^2^ g^−1^]	[cm^3^ g^−1^]	[cm^3^ g^−1^]	C	N	O	[F g^−1^]
untreated (NC‐900*)^[g]^	1233	0.44	2.11	90.8	6.6	2.6	117
NC‐0C‐1h	–	–	–	83.8	6.3	9.9	138
NC‐0C‐2h	–	–	–	84.1	6.0	9.9	156
NC‐0C‐4h	849	0.31	1.48	80.5	6.8	12.7	168
NC‐0C‐7h	–	–	–	83.0	5.4	11.6	135
NC‐20C‐1h	1197	0.41	2.01	81.6	5.8	12.6	158
NC‐20C‐2h	–	–	–	82.2	6.3	11.5	148
NC‐20C‐4h	–	–	–	77.4	5.2	17.4	120
NC‐20C‐18h	–	–	–	77.7	4.6	17.7	69

[a] The sample name denotes the treatment temperature and time. [b] Specific surface area as determined by BET2 analysis of N_2_ desorption at 77 K. [c] Micropore volume calculated by the Dubinin–Radushkevich method. [d] Total pore volume filled up to a relative pressure (*P*/*P*_0_) of 0.99. [e] Near‐surface elemental composition obtained from XPS. [f] Specific capacitance measured at a scan rate of 5 mV s^−1^. [g] The acid‐treated NC‐900* was from another batch, relative to Table 1.

The rise and fall of capacitance versus treatment time indicates that two opposing factors are at play. To understand these factors, we must first understand how microstructure and surface chemistry change with the surface treatments. First, let us consider EDLC, which depends strongly on surface area and pore structure. These were studied using N_2_ sorption porosimetry at 77 K (Table 2), combined with two‐parameter Brunauer–Emmett–Teller (BET2) analysis of the specific surface area and Dubinin–Radushkevitch analysis of the micropore volume. Interestingly, the surface treatments decreased the carbon's SSA (by as much as 30 % when treated at 0 °C). The main effect of the treatment was a decrease in the micropore volume. This could reflect the blocking of micropores by newly grafted surface functionalities^36^, ^39^ and/or the etching of micropore walls, which turns them into mesopores.^40^ The second explanation, however, is hard to reconcile with the N_2_ sorption isotherm; the hysteresis in the isotherm, associated with mesopore content, does not increase (Figure S4). Thus, the most probable explanation for the decrease in SSA and in micropore volume is the blocking of pores by new surface groups. Importantly, the improvement in capacitance cannot be attributed to an increase in EDLC charge storage because the SSA actually decreases. Therefore, PC contributes at least 45 % of the capacitance of NC‐0C‐4h—the entire rise effected by the treatments.

To explain this boost in capacitance, we examined the changes to the surface chemistry during the treatment. The oxygen/carbon ratio increased with treatment time (Table 2), indicating surface oxidation. The oxygen content reached saturation without any change to the shape of the O 1s X‐ray photoelectron spectroscopy (XPS) peak (Figure S5). This suggested that the oxygen‐based functional groups saturated the surface. Beyond this point, further treatment led to carbon deterioration as observed from the total disappearance of carbon after prolonged/hot oxidations.

**Figure 4:**
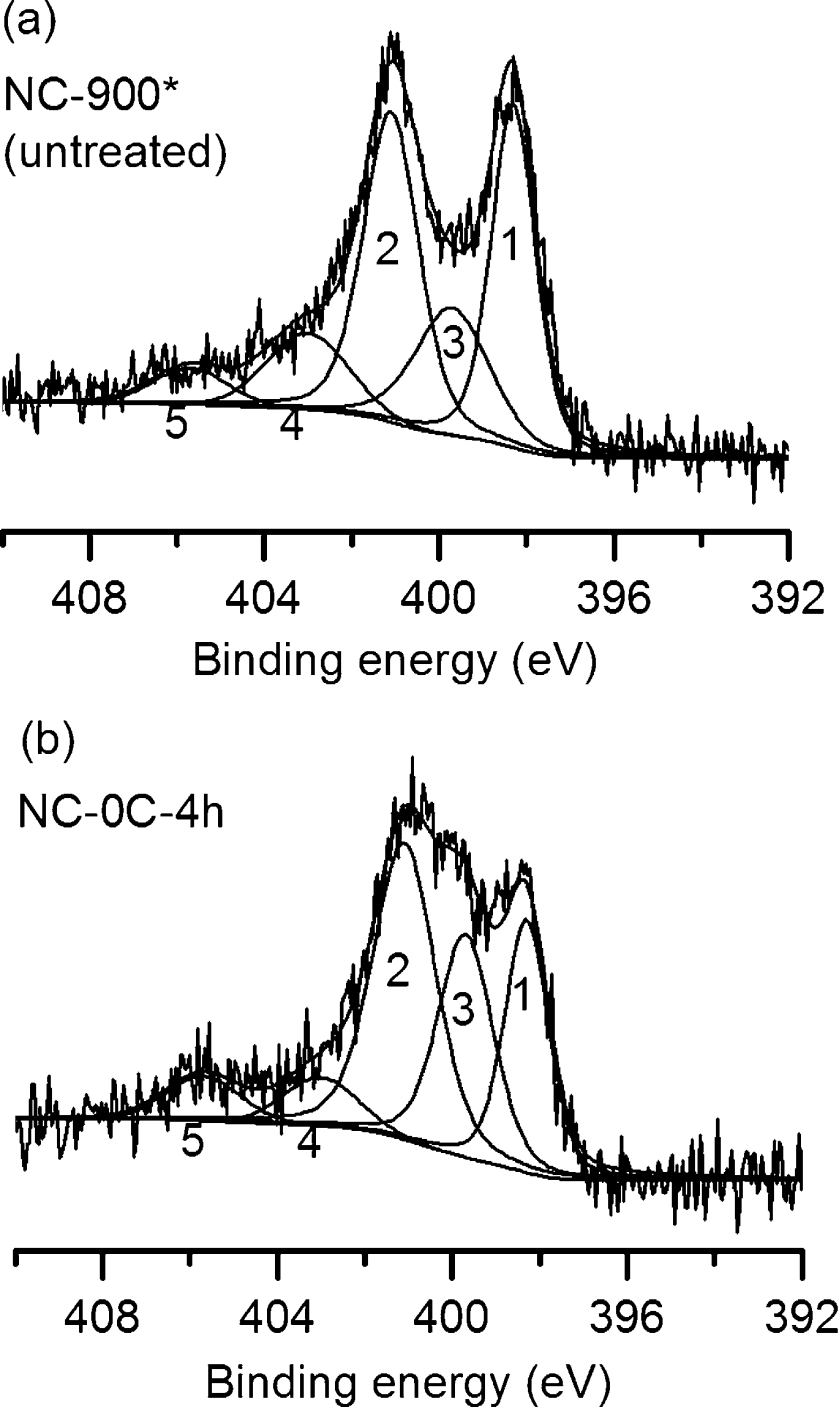
XPS in the N 1s region of (a) untreated carbon (NC‐900*), and (b) carbon treated at 0 °C for 4 h. The spectrum was fitted with peaks representing different nitrogen functionalities, including 1—pyridinic, 2—graphitic, 3—pyrrolic/pyridonic, 4—“oxidized”, and 5—nitro groups.

To study how the acidic treatment affects the distribution of nitrogen functionalities in the carbon, XPS spectra of the N 1s region were mathematically deconvoluted and their areas were compared (Table 2). The deconvoluted spectra for the untreated carbon and the best‐treated carbon are presented in Figure 4. The nitrogen dopants in these carbons include graphitic (401.1 eV), pyridinic (398.3 eV), and pyridonic/pyrrolic nitrogen atoms (399.7 eV); various oxidized nitrogen atoms (402–404 eV); and nitro groups (405.7 eV).^41^

**Figure 5:**
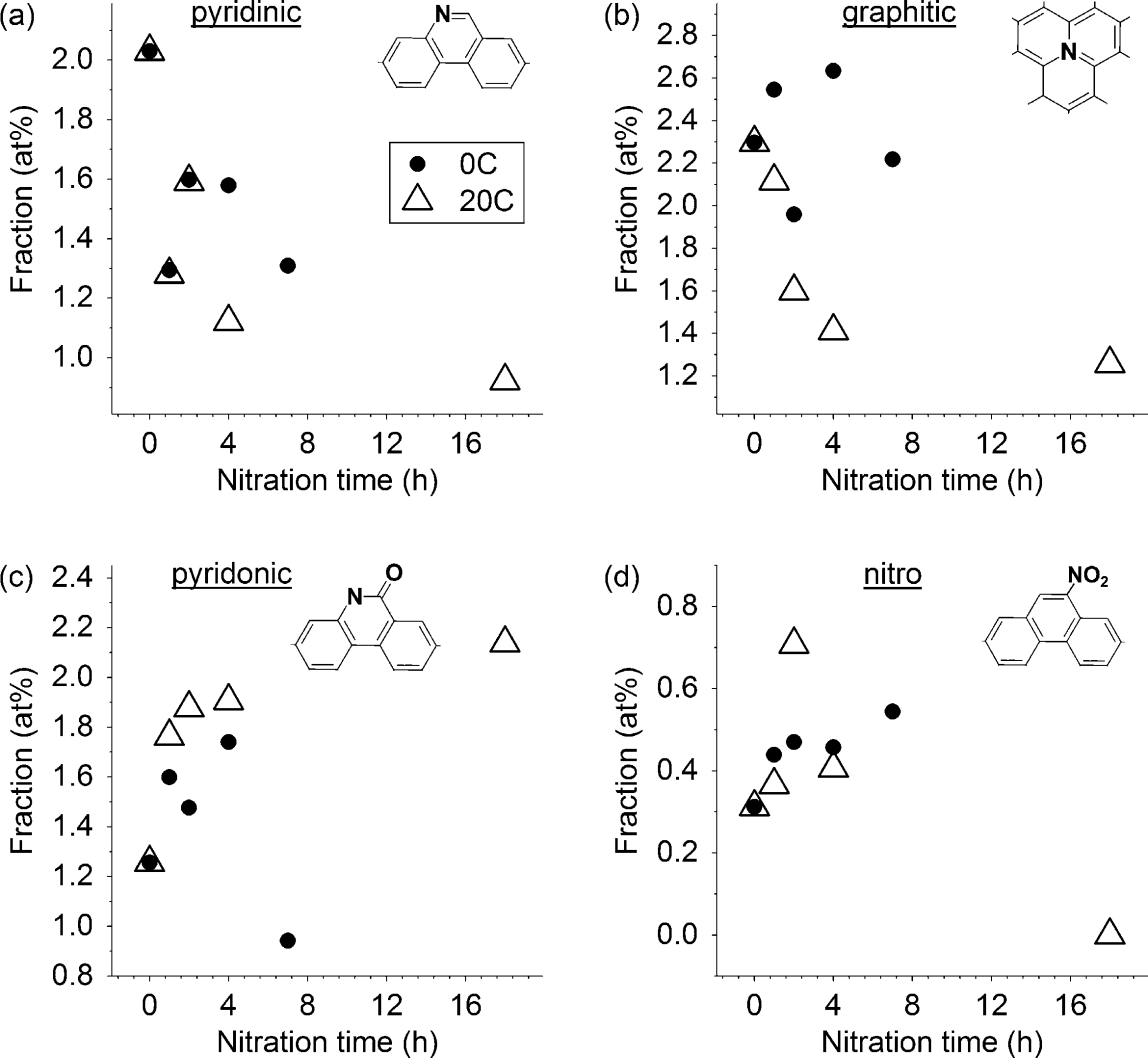
Changes in the total atomic fraction of different nitrogen functionalities as a function of treatment time. Two treatment temperatures were explored: 0 °C (solid circles) and 20 °C (empty triangles), and four functionalities as follows: (a) pyridinic nitrogen, (b) graphitic nitrogen, (c) pyridonic nitrogen, (d) nitro groups. The atomic fraction is derived from XPS fitting and integration.

The evolution of nitrogen content with treatment time is shown in Figure 5. The most common nitrogen functionalities were graphitic and pyridinic, which were present in all pyrolysis‐derived carbons. Both of these groups decrease in relative intensity throughout the treatments (Figure 5 a and b), from approximately 2.0 at % to 1.0 at %. This decrease was the most pronounced and smooth in the 20 °C treatments. In contrast, only pyridinic nitrogen was removed in the 0 °C treatment, whereas the graphitic nitrogen fraction remained roughly constant (2.0–2.6 at %). This process was attributed to the etching, as discussed earlier. A warmer and/or prolonged treatment is more effective in etching, as seen earlier in the saturation values of the oxygen/carbon ratio. Pyridinic nitrogen atoms at plane edges (Figure 1) were the first to leave. Hence, even the colder treatment was sufficient for their removal.

**Figure 6:**
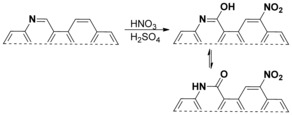
Two possible surface reactions that occur when nitrogen‐doped carbon is treated with HNO_3_/H_2_SO_4_: Hydroxylation of a carbon vicinal to a pyridinic nitrogen (yielding a pyridonic group) and nitration (yielding a nitro group).

Etching was not the only process driving the decrease in the pyridinic nitrogen fraction. Oxidation of the vicinal carbon atoms formed a pyridonic group (Figure 6) with higher N 1s binding energies. The increase in amount of this group throughout the 20 °C treatment is evident in Figure 5 c. In the 0 °C treatment, the pyridonic nitrogen fraction also increases and finally drops. This drop coincides with a drop in the total nitrogen fraction and in specific capacitance after prolonged treatment. The assignment of the 399.7 eV peak is often challenging^41^ as this is the binding energy of both pyridonic and pyrrolic nitrogen atoms. However, pyrrolic groups cannot form by treatment at room temperature.^42^ Thus, the increase in this peak was assigned to the formation of pyridonic nitrogen atoms by the oxidation of a carbon atom vicinal to a pyridinic nitrogen atom.

Interestingly, the pyridonic groups were not the only new groups resulting from the acid treatment. The new peak at 405.7 eV revealed the formation of nitro groups (Figure 6). This is in agreement with previous studies, in which nitric/sulfuric acid treatments led to pronounced nitro peaks in the XPS spectra.^43^, ^44^ These groups grew steadily in relative fraction during the 0 °C treatment and passed through a maximum during the 20 °C treatment (Figure 5 d). This indicates that the nitro groups were not the most stable oxidation products and suggested that optimization of the treatment time is crucial for obtaining the best conductivity.

Pyridinic, pyridonic, nitro, and pyrrolic nitrogen atoms have all been discussed as possible pseudocapacitive sites in doped carbons.^6^, ^7^, ^8^, ^9^, ^19^, ^20^, ^21^, ^22^, ^23^ Even though assigning the increase in capacitance to specific functional groups is difficult, it is nonetheless clear that nitrogen‐ and nitrogen–oxygen groups take part in pseudocapacitive charge storage through a variety of mechanisms (Figure 1).

## Conclusions

In this work, we demonstrated that nitrogen‐doped, hierarchically porous carbons derived from Mg‐based metal–organic frameworks (MOFs) can be used as electrochemical capacitors. The structure and surface chemistry of these carbons can be optimized orthogonally. Selecting an optimal pyrolysis temperature, or combining a pyrolysis process with a second thermal process, is crucial for identifying an optimal combination of surface area, pore structure, and dopant concentration. Relative to a typical one‐step pyrolysis at 700 °C (specific capacitance 62 F g^−1^), the two‐step heat treatment (900 °C/washing/1000 °C) nearly doubled the specific capacitance (117 F g^−1^). Next, treating the carbon with a 1:4 mixture of nitric and sulfuric acid introduced a variety of oxygen‐ and nitrogen‐oxygen surface functionalities. These groups boosted the pseudocapacitive charge storage mechanisms, increasing the capacitance up to 168 F g^−1^—over 40 % improvement just from the treatments and nearly 200 % relative to the original carbon. The effect of the surface treatment was complex, including etching out pyridinic and graphitic nitrogen atoms while introducing new functional groups such as pyridonic and nitro groups. The density of the pyridonic groups reached saturation as the treatment progressed, whereas the specific capacitance peaked and then dropped. The positive effect of the surface functionalities was eventually outweighed, and capacitance declined. This may reflect the blocking of micropores by functional groups^39^ (less electrochemical double‐layer capacitance) or a decrease in conductivity caused by oxygenation.^45^, ^46^

## Experimental Section

### Materials and instrumentation

Unless stated otherwise, chemicals were purchased from either Sigma–Aldrich or Alfa Aesar, and used without further purification.

N_2_ adsorption–desorption isotherms were measured on a Thermo Scientific Surfer instrument at 77 K, using vacuum‐dried samples (200 °C/3 h). Isotherms were analyzed by the ThermoFischer Advanced Data Processing 6.0 software, using the BET2 model for specific surface area, the Dubinin–Radushkevitch model for micropore volume.

The XPS measurements were performed using a PHI VersaProbe II scanning XPS microprobe (Physical Instruments AG, Germany). Analysis was performed using a monochromatic AlK_α_ X‐ray source with a power of 24.8 W and a beam size of 100 mm. The spherical capacitor analyzer was set at a 45° take‐off angle with respect to the sample surface. The pass energy was 46.95 eV, yielding a full width at half maximum of 0.91 eV for the Ag 3d_5/2_ peak. Peaks were calibrated using the C 1s position. Curve fitting was performed using XPSPeak 4.1.

### Procedure for carbon synthesis

Nitrogen‐doped carbon was prepared using the method reported previously.^25^, ^26^, ^27^ Briefly, nitrilotriacetic acid (N(CH_2_COOH)_3_; NTA) was mixed with basic magnesium carbonate ((MgCO_3_)_4_Mg(OH)_2_) at 1:1 Mg/NTA ratio in deionized (DI) water at 85 °C. The solid was precipitated by slowly adding an excess of ethanol and then chilling in an ice bath for 2 h. The resulting white paste was scraped out, vacuum dried at 40 °C for 48 h, and ground in an automated grinder. The resulting hard white powder was pyrolyzed in argon at temperatures of 700–1000 °C (samples denoted herein as NC‐700 to NC‐1000, respectively). The MgO nanoparticles were washed by stirring overnight in 0.5 m citric acid. The carbons were dried at 130 °C. In some cases, the carbons were further heat‐treated in argon to 1000 °C/1 h.

### Procedure for surface treatment

Surface treatments were performed by adding 10 mL of H_2_SO_4_ (96 %, 178 mmol) to 80 mg of NC‐900* in a 25 mL round‐bottom flask. This mixture was stabilized at the desired temperature (0 or 20 °C). Subsequently, 2.5 mL of HNO_3_ (65 % aq., 37 mmol) was added in ten portions while stirring over 15 min. The resulting suspension was stirred for the indicated time (1–18 h). The reaction was quenched by pouring the reaction mixture in 300 mL ice water and washed on a mobile phase filtration setup using a nylon filter (0.45 μm pore size) with 1 L of DI water. The sample was dried at 80 °C for 24 h.

### Electrochemical experiments

Electrodes were prepared using a 80:10:10 mass ratio of carbon sample/PTFE/carbon black (PTFE=polytetrafluoroethylene). In a typical preparation, the carbon sample (16 mg) was mixed with carbon black (2 mg, 50 % compressed) in a mortar. PTFE (2.2 μL, 60 % suspension in water, 2 mg PTFE) was then added with water (75 μL). This slurry was ground in a mortar and pressed to a film. The films were then cut into squares of roughly 1 cm^2^. The working electrode consisted of carbon films pressed between a 0.125 mm tantalum plate and Whatman filter paper using the electrode press. The layers were pressed together in a specialized testing clamp, 3D‐printed from high impact polystyrene (Figure S6). Cyclic voltammetry experiments were performed on a Gamry Instruments Reference 600 potentiostat using a three‐electrode setup. A SCE was used as a reference in a three‐electrode cell, with a graphite rod serving as the counter electrode.

## Conflict of interest

*The authors declare no conflict of interest*.

## Supporting information

As a service to our authors and readers, this journal provides supporting information supplied by the authors. Such materials are peer reviewed and may be re‐organized for online delivery, but are not copy‐edited or typeset. Technical support issues arising from supporting information (other than missing files) should be addressed to the authors.

Supplementary
