# Supplementary material for: Boosting the Supercapacitance of Nitrogen‐Doped Carbon by Tuning Surface Functionalities
Source: ChemSusChem. 2017 Aug 15;10(20):4018–24. doi: 10.1002/cssc.201700902 (PMC5724695; doi:10.1002/cssc.201700902)
Supplement: Supplementary file 1 — Supplementary [file CSSC-10-4018-s001.pdf]

CHEMISTRY & SUSTAINABILITY
CHEMSUSCHEM
ENERGY & MATERIALS

Supporting Information

Boosting the Supercapacitance of Nitrogen-Doped Carbon by Tuning Surface Functionalities

Jasper Biemolt+,[a] Ilse M. Denekamp+,[a] Thierry K. Slot,[a] Gadi Rothenberg,*[a] and David Eisenberg*[a, b]

cssc_201700902_sm_miscellaneous_information.pdf

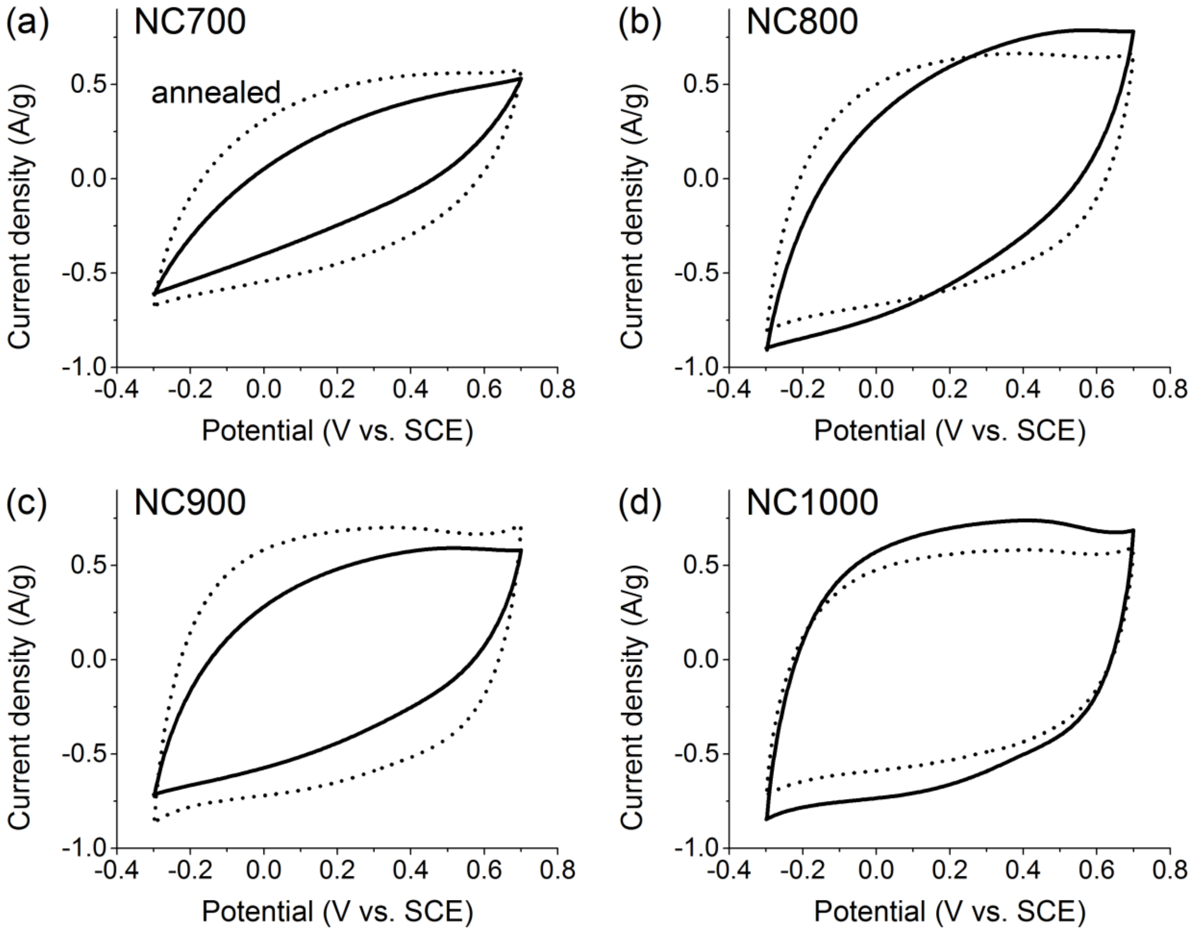

Figure S1. Cyclic voltammetry in 1 M H_2SO_4 at 5 mV/s, for carbons before a 1000 °C heat treatment (solid line) and after it (dotted line).

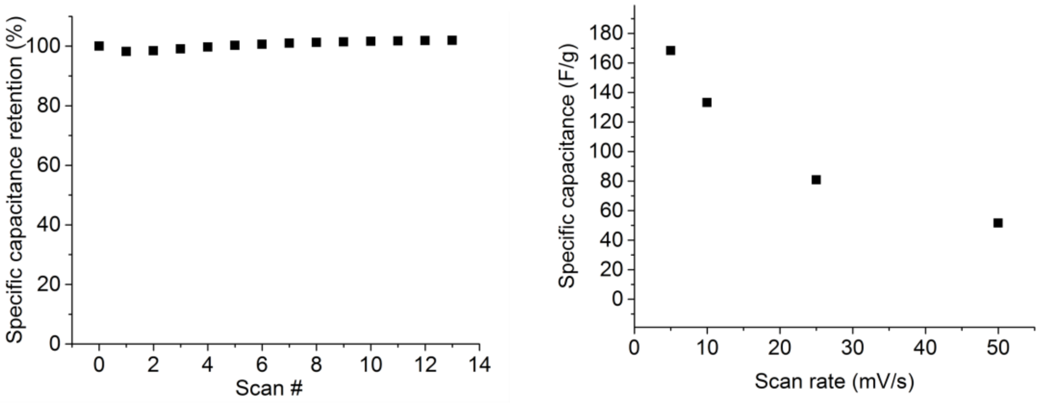

Figure S2. (a) Cycling behavior and (b) rate capability of sample NC-0C-4h in 1M H_2SO_4.

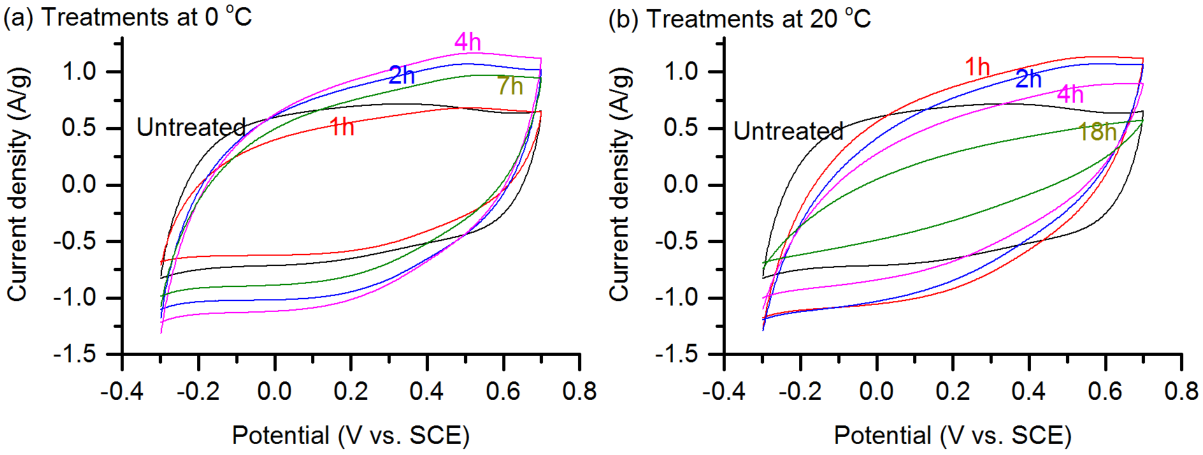

Figure S3. Cyclic voltammetry in 1 M H_2SO_4 at 5 mV/s, for carbons from the NC-900* batch, treated at (a) 0 °C and (b) 20 °C. Different treatment times are marked on the scheme.

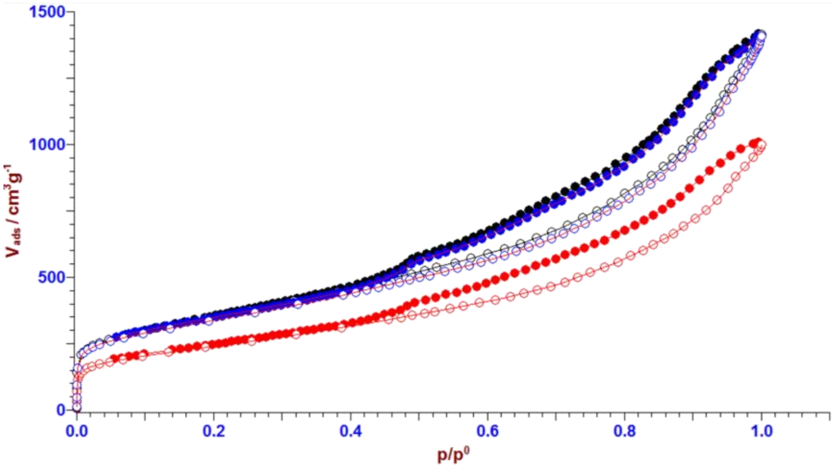

Figure S4. N_2 adsorption-desorption isotherms at 77K on carbon NC900* (black), and carbons treated in acid at 0 °C / 4h (red) and at 20 °C / 1h (blue).

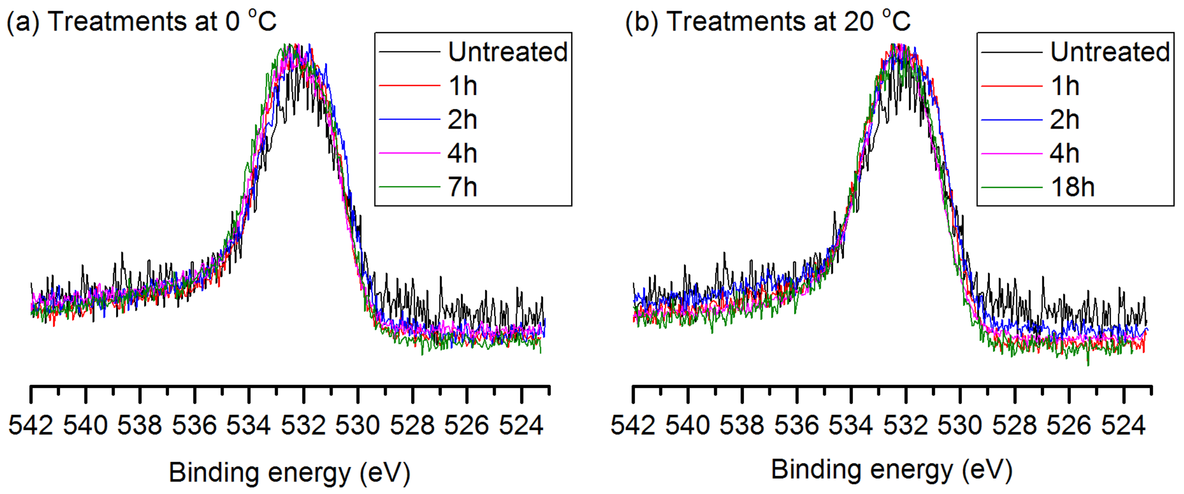

Figure S5. X-ray photoelectron spectra in the O-1s region for carbons treated at (a) 0 °C and (b) 20 °C.

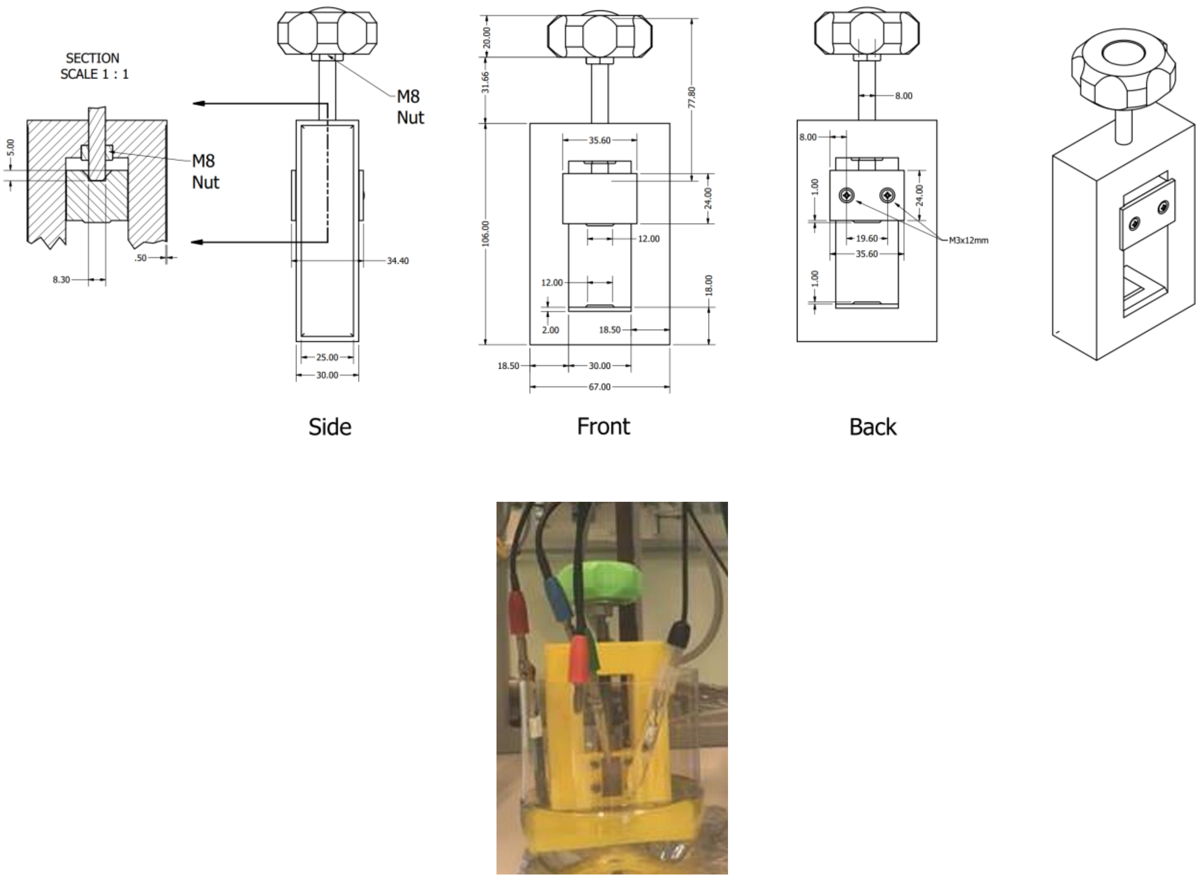

Figure S6. CAD model and final structure of a supercapacitor testing device, 3D-printed from high impact polystyrene.
